# Neostriatal Neuronal Activity Correlates Better with Movement Kinematics under Certain Rewards

**DOI:** 10.3389/fnins.2016.00336

**Published:** 2016-08-05

**Authors:** Ioan Opris, Mikhail A. Lebedev, Randall J. Nelson

**Affiliations:** ^1^Miami Project, University of FloridaMiami, FL, USA; ^2^Department of Neurobiology, Duke UniversityDurham, NC, USA; ^3^Department of Anatomy and Neurobiology, The University of Tennessee Health Science CenterMemphis, TN, USA

**Keywords:** basal ganglia, neostriatum, neuronal activity, movement preparation, hand movement, multilinear regression, reward uncertainty, motivation

## Abstract

This study investigated how the activity of neostriatal neurons is related to the kinematics of movement when monkeys performed visually and vibratory cued wrist extensions and flexions. Single-unit recordings of 142/236 neostriatal neurons showed pre-movement activity (PMA) in a reaction time task with unpredictable reward. Monkeys were pseudo-randomly (75%) rewarded for correct performance. A regression model was used to determine whether the correlation between neostriatal neuronal activity and the kinematic variables (position, velocity, and acceleration) of wrist movement changes as a function of reward contingency, sensory cues, and movement direction. The coefficients of determination (CoD) representing the proportion of the variance in neuronal activity explained by the regression model on a trial by trial basis, together with their temporal occurrences (time of best regression/correlation, ToC) were compared across sensory modality, movement direction, and reward contingency. The best relationship (correlation) between neuronal activity and movement kinematic variables, given by the average coefficient of determination (CoD), was: (a) greater during trials in which rewards were certain, called “A” trials, as compared with those in which reward was uncertain called (“R”) trials, (b) greater during flexion (Flex) trials as compared with extension (Ext) trials, and (c) greater during visual (VIS) cued trials than during vibratory (VIB) cued trials, for the same type of trial and the same movement direction. These results are consistent with the hypothesis that predictability of reward for correct performance is accompanied by faster linkage between neostriatal PMA and the vigor of wrist movement kinematics. Furthermore, the results provide valuable insights for building an upper-limb neuroprosthesis.

## Introduction

A significant proportion of neurons from neostriatum (NS) modulate their firing rate in response to environmental stimuli and/or fire before movement initiation and/or execution (Aldridge et al., 1980; Liles, 1985; Schultz and Romo, 1988; Alexander and Crutcher, 1990; Kimura, 1990; Romo and Schultz, 1990; Gardiner and Nelson, 1992; Lebedev and Nelson, 1999; Opris et al., 2011a). Several studies indicate that NS neurons may also participate in associating task stimuli with rewards (Schultz and Romo, 1990; Apicella et al., 1991; Robbins and Everitt, 1996; Opris et al., 2009; Schultz, 2010) or in modulating movement vigor (Opris et al., 2011a). This connection between reinforcement and behavior may be mediated by the dopaminergic system at either sub-cortical or cortical levels (Robbins and Everitt, 1996; Schultz et al., 1997). After behaviors are learned, a state of expectation about the time of occurrence of reward delivery relative to requested behaviors is created (Apicella et al., 1991, 1992; Schultz et al., 1992, 1993).

It has also been suggested that during goal-oriented behavior NS neurons exhibit context-dependent behavioral responses that may vary depending on changes in the predictability of the linkage between stimuli and rewards (Schultz and Romo, 1990; Apicella et al., 1992; Schultz et al., 1992; Mirenowicz and Schultz, 1994). Moreover, several lines of evidence indicate that the NS neuronal activity may become better related to movements and sensory cues when behavioral conditions are novel (Mirenowicz and Schultz, 1994; Nelson et al., 1996; Berns et al., 1997). Sensitivity to novelty seems to be a property of neurons in the substantia nigra, the ventral striatum, and the prefrontal cortex (Apicella et al., 1991; Schultz et al., 1992, 1993; Watanabe, 1996; Berns et al., 1997; Schultz, 2010). Surmeier and Kitai (1997) indicated that the dopaminergic inputs to neostriatum may “shape” the activity of NS neurons involved in motor behavior. Therefore, the substantia nigra, ventral striatum and prefrontal cortex may influence segments of the so-called “motor loop” (which includes the dorsal striatum) where pre-movement activity (PMA) often occurs (Alexander et al., 1986; Hoover and Strick, 1993; Graybiel et al., 1994; Watanabe, 1996; Berns et al., 1997).

We hypothesize that primate dorsal striatum (Putamen and Caudate Nucleus) is part of a system that modulates the behavioral parameters (i.e., movement kinematics: position, speed, and acceleration), depending on the probability of the expected reward (Fu et al., 1995; Fiorillo et al., 2003; Lee and Assad, 2003; Stark et al., 2007; Pekny et al., 2015; Reppert et al., 2015). Dorsal striatum may be responsible for enhancing movement vigor when rewards are certain and decreasing the vigor when rewards become uncertain (Turner and Desmurget, 2010; Opris et al., 2011a). This hypothesis is supported by the finding that changes in dorsal striatal activity occur shortly after go cues and clearly earlier than the movements (100–200 ms before movements). Therefore, *certain rewards* may mediate a stronger correlation (Opris et al., 2011a) between neostriatal PMA and the kinematics of wrist movements.

Below, we describe the changes in NS neuronal activity in relation to movement kinematics by using a multilinear regression analysis (Ashe and Georgopoulos, 1994). Furthermore, we believe that striatal modulations can provide a good motor signal for neuroprosthetics (Lebedev and Nicolelis, 2006; Nicolelis and Lebedev, 2009). The goals of this study was to: (a) determine if the correlation between NS firing rates and the movement kinematics varies as a function of go-cue modality, movement directions, behavioral contexts; and (b) quantify the occurrence and timing of best relationship/correlation between NS neuronal activity and hand movement kinematics. Moreover, since changes in movement related NS activity often occur 100–200 ms before movements, it is possible that unpredictable behavioral conditions modify NS activity that is involved in movement preparation and initiation. The results are relevant for the decoding of wrist kinematics for building an upper-limb neuroprosthesis (Lebedev et al., 2005; Aggarwal et al., 2009).

## Materials and methods

### Experimental apparatus and behavioral paradigm

Two adult male rhesus monkeys (*Macaca mulatta*: E, N) were trained to make wrist flexion and extension movements in response to vibratory or visual stimuli (Lebedev and Nelson, 1995; Liu et al., 2005; Opris et al., 2011a). The monkeys were cared for in accordance with the National Research Council Guide for the Care and Use of Laboratory Animals. Experimental protocols were approved by the Animal Care and Use Committee of The University of Tennessee Health Science Center, Memphis. Detailed descriptions of the experimental apparatus have been provided elsewhere (Gardiner and Nelson, 1992; Lebedev and Nelson, 1995; Liu et al., 2005, 2008). A brief description is provided below.

**Figure 1:**
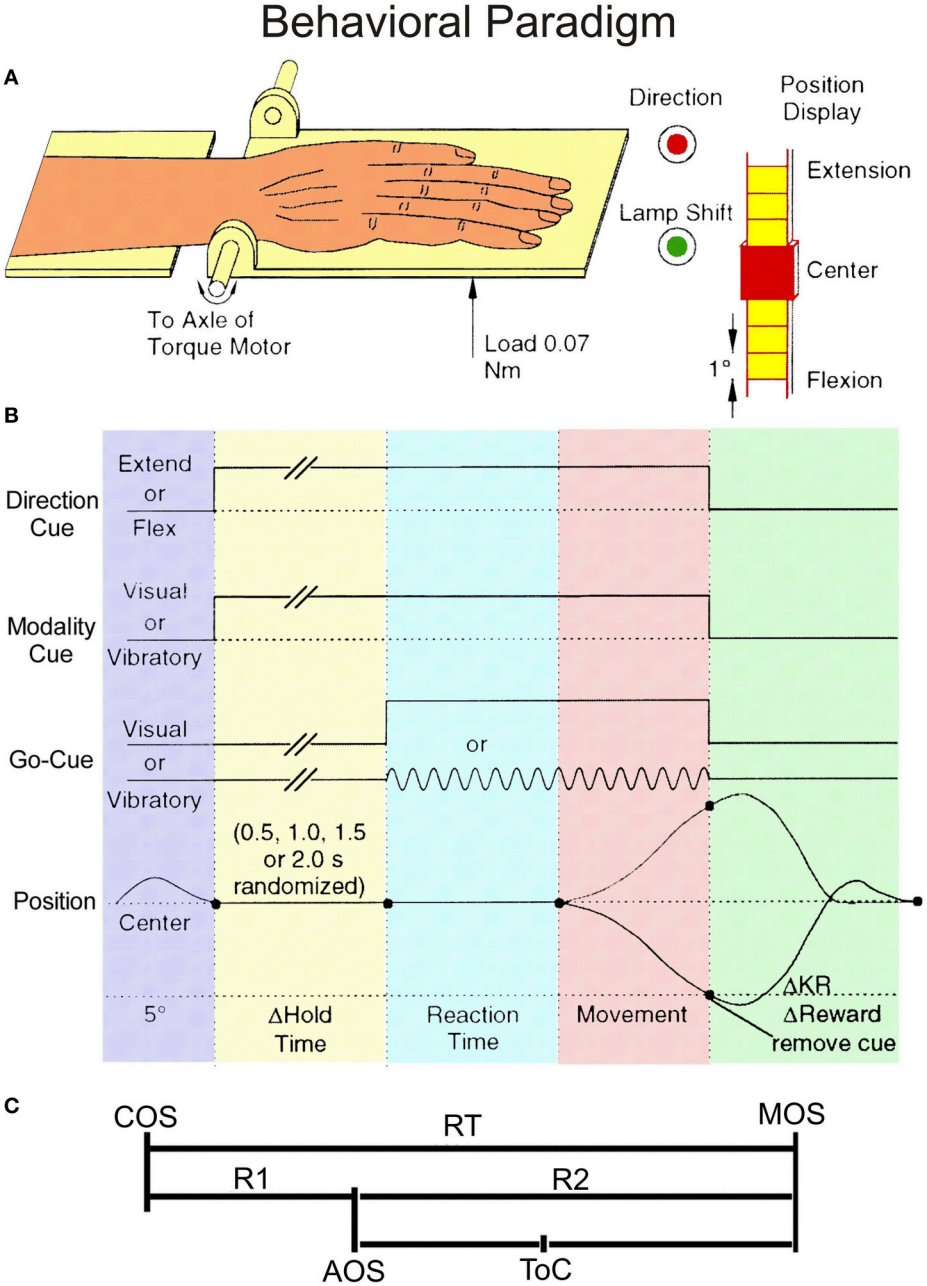
**Behavioral paradigm. (A)** The experimental setup showing monkey's hand on the handle together with the instruction LEDs. **(B)** Schematic diagram of the unpredictable task. The direction cue was given by a red LED that was illuminated during extension trials, but not during flexion trials. The modality cue was a green LED that was illuminated during vibratory cued trials but not during visually cued trials. The onset of these instructional cues was coincident with the onset of the hold period. They remained lit until the end of the trial, coincident with reward delivery. Go-cues that signaled the monkeys could initiate wrist movements were presented after a variable time delay of 0.5, 1.0, 1.5, or 2.0 s (pseudo-randomized). **(C)** Divisions of the Reaction Time (RT) interval. RT has been split into two intervals: R1, the latency from cue onset (COS) to premovement activity onset (AOS), and R2, the time from AOS until movement onset (MOS). ToC represents the time of best correlation (time at best CoD).

Each monkey sat in an acrylic monkey chair, with its right palm on a movable plate (Figure 1A). One end of the plate was attached to the axle of a brushless D.C. torque motor (Colburn and Evarts, 1978). A load of 0.07 Nm was applied to the plate. The load assisted wrist extensions and opposed wrist flexions. Feedback of current wrist position was provided by a visual display consisting of 31 light-emitting diodes (LEDs), located 35 cm in front of the animal. The middle, red LED corresponded to a centered wrist position. Yellow LEDs (above and below the middle LED) indicated successive angular deviations of 1°. Two instructional LEDs were located in the upper left corner of the visual display. When the first red LED was illuminated at the start of a trial, it indicated that extension movements should be made; otherwise flexions were required. When the second, green LED was illuminated, it informed the monkey that the go-cue for that trial would be a palmar vibration; otherwise, the go-cue was the illumination of one of two LEDs which were each 5° from the center. Vibratory go-cues of 27, 57, and 127 Hz were routinely used. In this paper we consider only records of neuronal activity triggered by vibratory cues at 57 Hz and/or visual go-cues.

The behavioral paradigm is illustrated schematically in Figure 1B and the partition of reaction time (RT) interval in Figure 1C. Each trial began when the monkey centered the plate. At this time a movement direction request was given by the instructional LED as described above. The monkey was required to hold the plate in the centered position for 0.5, 1.0, 1.5, 2.0 s (pseudo-randomized) without moving, until a go cue (visual or vibrotactile) was presented. If the animal moved prior to the completion of the hold period, the trial was canceled. If the monkey made the requested movement of 5° or greater, he received a fruit juice reward. The reward delivery for blocks of 10 trials per direction was pseudorandom with correct performance being rewarded on average 75% of the time. Two unrewarded trials were never imposed sequentially. The type of trials under this pseudorandom reward schedule included: (i) rewarded trials, for which the current and the immediately preceding trial were rewarded, called regular (“R”) trials, (ii) unrewarded trials, and (iii) trials immediately following withheld rewards, called after (“A”) trials or *certain reward* trials (while reward in all other trials was *uncertain*). In some instances there were trials in which the animal failed to perform properly (i.e., made a movement in the wrong direction). For analyses we required that each neuron must have at least 4 valid trials per condition. If any single group of records had fewer than 4 trials, the data from that group were not included in the analyses.

### Electrophysiological recordings and histology

**Figure 2:**
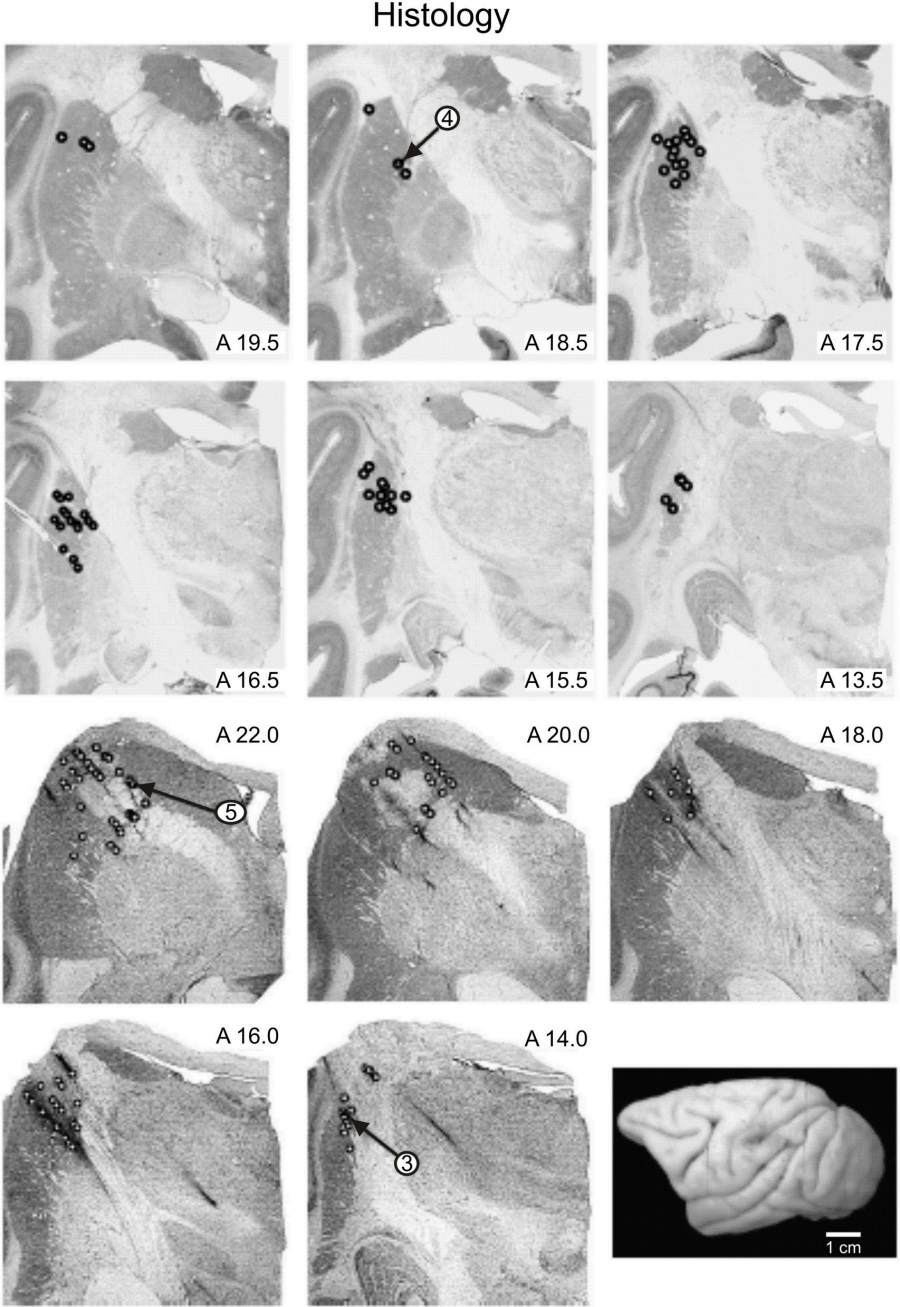
**Reconstructed locations of recorded neurons**. Location of recorded neurons from monkeys E (panels in the upper row) and N (panels in the lower row) are shown. Small open circles indicate the locations in the basal ganglia at which records were taken. The recording locations of cells whose records have been illustrated are indicated by the arrows. A drawing of the dorsolateral surface of monkey E is shown in the lower right panel.

Once an animal reached a stable daily performance level (~2000 rewarded trials per experimental session), it was prepared for recording. A stainless steel recording chamber was surgically implanted over the skull to allow for extracellular recordings of the activity of basal ganglia neurons by using platinum-iridium microelectrodes with impedances of 1–2 MOhms (see Nelson et al., 1991; Gardiner and Nelson, 1992). Transdural penetrations began no sooner than 1 week after the chamber implantation. In each recording session, a microelectrode was lowered into the striatum and the activity of single units was amplified, discriminated, and stored in a computer by conventional means (Lebedev and Nelson, 1995; Liu et al., 2005). Neuronal receptive fields (RFs) were examined by lightly touching punctuate skin surfaces, manipulating joints, and palpating muscles. On occasion, the electromyographic (EMG) activity patterns of forearm muscles acting across the wrist were recorded using intramuscular EMG wires (see Nelson, 1987). EMG activity was converted into pulse data (Nelson, 1987; Vaadia et al., 1988). On the last recording day, electrolytic lesions were made to mark some recording locations by passing 10 μA of current for 10–20 s. These lesions provided references for the histological reconstruction of the recording sites. The animal was then deeply anesthetized with sodium pentobarbital and transcardially perfused with 10% buffered formol-saline. The brain was removed from the skull, and cut on a freezing microtome into 50 μm thick coronal sections. Histological sections of the basal ganglia (Figure 2) were stained for Nissl substance. Recording sites were reconstructed based on the depth of each electrode penetration and its location with respect to the marking lesions (Nelson, 1988; Nelson et al., 1991).

### Data analysis

Neuronal activity data, recorded on-line (Lebedev and Nelson, 1995, 1996; Liu et al., 2005; Opris et al., 2011a), were processed by off-line analysis programs and displayed as rasters, peri-event time histogram (PETH), fractional interspike interval (ISI) histogram (FISIH; Ashe and Georgopoulos, 1994), and traces of position, velocity, and acceleration, aligned on important events in the animal's behavior. A multilinear regression analysis (Ashe and Georgopoulos, 1994) has been used to regress low-pass filtered single trial spike functions coming from a FISIH, against position, velocity, and acceleration traces.

### Multilinear regression analysis

An important step in our implementation of the regression analysis is the formulation of a FISIH from single trial neuronal activity records. We followed almost precisely the published methods (Ashe and Georgopoulos, 1994; Taira et al., 1996). This type of histogram is a filtered version of a binned PETH, in which the fraction of each ISI that is distributed across a given bin is calculated. The whole ISI is equal to unity, whereas each bin receives fractional contributions relative to the interval of that bin. We have employed single trial FISIHs, as well as, composite FISIH representing up to 40 trials. Each spike train was binned in 5 ms bins and the time-varying frequency of cell discharge, d(t), was computed from FISIHs. The resulting spike function, f(t), was smoothed using a 5-point low-pass filter, then the squared root of each point comprising the function was taken (Cox and Lewis, 1966; Ashe and Georgopoulos, 1994) and the resultant was divided by the binwidth (bw; in seconds), to convert this function to units proportional to the instantaneous firing rate:

(1) $$\begin{array}{l} \text{f}\left(\text{t}\right)=\text{sqrt}\left(\text{d}\left(\text{t}\right)\right)∕\text{bw} \end{array}$$

The position trace was smoothed using a single 5-point low-pass filter while the velocity and acceleration were obtained from the smoothed position trace as single or double discrete time derivatives.

**Figure 3:**
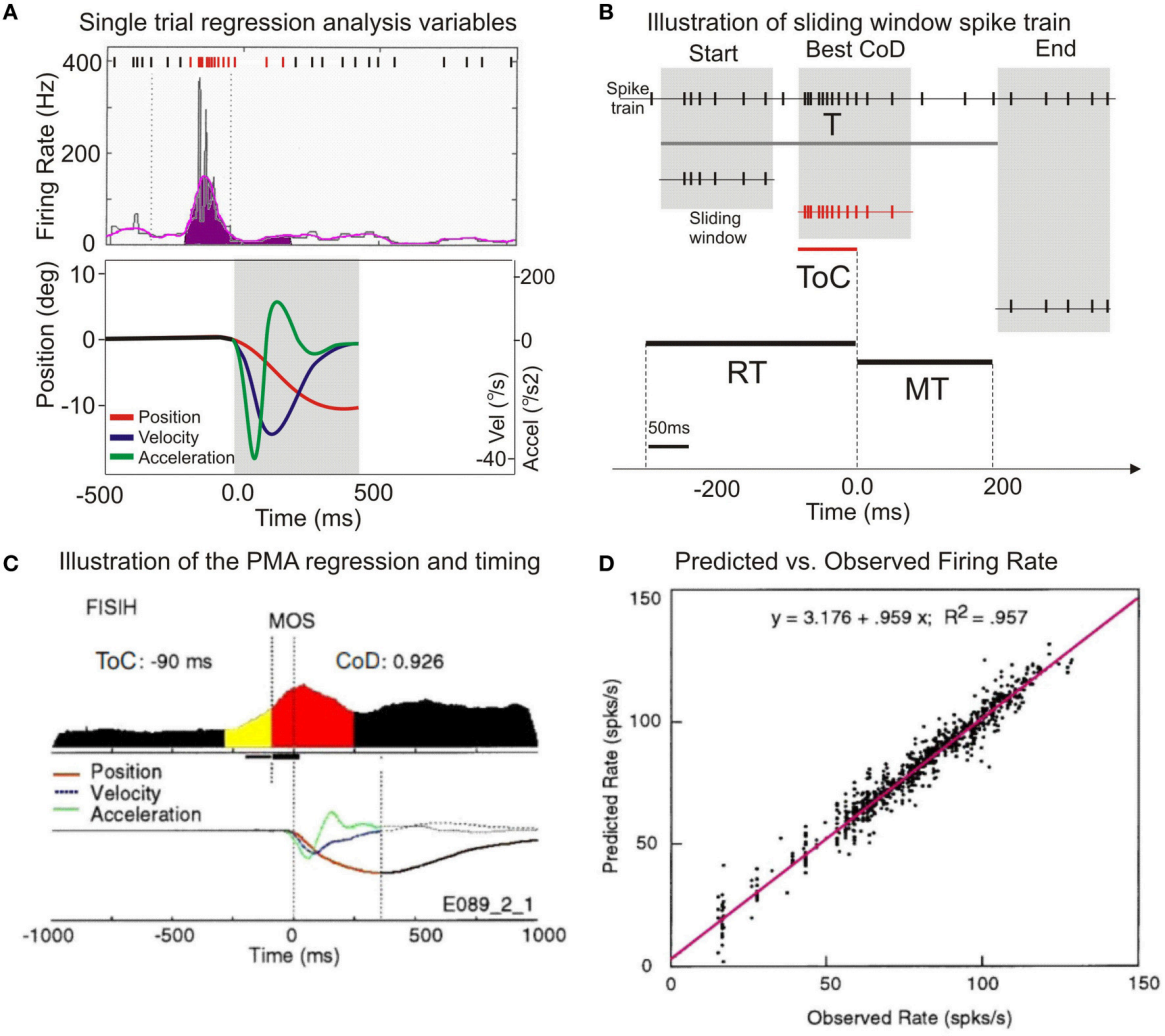
**Illustration of the application of multilinear regression analysis. (A)** A single trial histogram of the neuronal activity expressed as mean firing rate (in spikes/s), together with the kinematic variables of position, velocity, and accelaration. **(B)** Illustration of the sliding window regression analysis. RT, reaction time; MT, movement time; ToC is the time of best correlation (at best CoD). **(C)** A typical putamen neuron with increased premovement activity during vibratory cued trials is shown. The display of the average kinematics for uncertain reward flexion trials R, are centered on MOS, on both panels. **(D)** The scatter diagram of the predicted discharge rate plotted against the observed rate. Regression equation of predicted vs. observed mean firing rates and the regression coefficient in the upper part of the panel.

A multilinear regression algorithm (Sokal and Rohlf, 1981) was applied to epochs of single trials so that portion of the single trial FISIH was expressed as a function of the animal's hand position, velocity, and acceleration during the initial phase of the movement. The regression analysis compares, on a trial-by-trial basis (Figure 3A), the frequency of cell discharge, f, at time t_0_+τ, where τ is the variable time shift of a “sliding window” (Figure 3B), relative to movement onset, and the duration of t_0_ is equal to the movement time. The window was advanced in time steps of 5 ms over the behaviorally important epoch T (the number of steps being T/bw). The variable T is defined as the interval that begins 50 ms after the cue onset, and ends at the time at which the movement reached amplitude of 5° from center. The square root of the discharge frequency, f, at time t_0_+τ, was expressed as a function of the position (°), velocity (°/sec), and acceleration (°/sec^2^) of monkey's hand at time t_1_, where t_1_ always began at movement onset (MOS). The regression equation is:

(2) $$\begin{array}{l} \text{f}({\text{t}}_{0}+\text{t})=\text{const}+\text{c1}\varphi \text{1}(\text{t1})+\text{c2}\varphi \text{2}(\text{t1})+\text{c3}\varphi \text{3}(\text{t1})\\ \;+\varepsilon ,{\text{t}}_{0}+\tau <\text{T and}|{\text{t}}_{0}|=|\text{t1}| \end{array}$$

where c1, c2, and c3 are the regression coefficients of position, velocity, and acceleration. The terms φ1(t1), φ2(t1) and φ3(t1) are the time-dependent variables of position, velocity, and acceleration, and ε is an error term. The coefficient of determination (CoD) is defined as the proportion of variance accounted for by the neuronal activity-to-movement correlation. Average CoDs were calculated only for valid trials having significant activity changes within the period of consideration.

The output of the regression analysis program provided averaged values of the CoD together with the time shift representing the time of best correlation (ToC) at which the best CoD occurred, as well as the partial regression coefficients at that point. The values of CoD ranged from 0 (the absence of correlation) to 1, where a full correlation of spike function and movement variables occurs. The analysis has been performed on single trial basis, but the results are displayed in normalized composite histograms for convenience and clarity (Figures 3A,C).

The changes in neuronal activity and their relationship to movement initiation and/or execution can be easily appreciated by visually estimating the outline of the composite histogram. FISIH patterns depict the activity of a cell, as indicated by the shape of the composite histogram and the site ToC relative to the shape of the composite histogram.

### Analysis of changes in neuronal activity

The changes of neuronal activity associated with wrist movement were analyzed using multilinear regression in single trial (Figure 3A). Data from a putamen neuron, recorded during vibratory cued “R” trials, is presented in Figure 3C. The baseline activity (Bkg) of each recorded neuron was calculated as its mean firing rate during the 250 ms prior to the presentation of go-cues, while the animal held his wrist in a centered position. The first change in the cumulative sum of more than three standard deviations (SDs), lasting for at least 40 ms, was designated as the activity onset (Onset or AOS). The total number of spikes occurring from AOS until movement onset (Figure 3C the yellow segment) divided by the interval divided by the number of trials was designated as the cell's pre-movement firing response (Resp). Mean firing rates of Bkg and Resp (4.9 and 24.7 spikes/s) together with the AOS (−147 ms) are further compared across cells and conditions (see **Figure 6B**). The period between AOS and MOS (R2) is defined in Figure 1C.

The regression analysis describes the covariant relationship between changes in NS activity and movement variables. The best covariance between activity and kinematics (cT) occurred ~90 ms before MOS. The average CoD of this cell was 0.926 and indicates that the neuronal activity has 92.6% of the variance explained by the multilinear regression analysis. This implies that the correlation between neuronal activity and the subsequent movement occurred at a time that proceeds the average onset time of electromyographic activity in this task (Gardiner and Nelson, 1992; Lebedev and Nelson, 1995). The time course of position, velocity, and acceleration are displayed beneath the FISIH; the regression analysis is described by Equation (2). To compare the actual firing properties with those predicted by the regression model, Figure 3D shows a scatter-gram of the predicted discharge rate plotted against the observed rate. Each column of dots is a trial, and each window shift is a dot. The plot indicates a consistent linear relationship between the predicted firing rate as calculated by the regression model and the rate recorded during experiments. This relationship suggests that the methods are reliable and compatible with those used by other investigators (Ashe and Georgopoulos, 1994; Taira et al., 1996).

### Quantitative criteria for PMA directionality

To determine the directionality of PMA, we calculated the difference between the Resp and the Bkg, and dubbed this difference the activity change (Act). In the cases in which Act was <1 spike/s, Act was recorded as 0 spike/s. If the sign of Act for a neuron differed as a function of movement direction, then that neuron was classified as “reciprocal” (encoding both flexions and extensions). In addition, we compared Act for flexion and extension movements and calculated their difference and sum. The ratio between difference and sum yields a scalar that should theoretically range between −1 and +1. Cases in which the ratio was −1 or +1, corresponded to those in which there was no change in activity for one direction of movement. These were coded as “directional.” Cases in which the scalar was > +1 or < −1 were instances in which PMA had the opposite sign as a function of movement direction, and were thus “reciprocal.” For those cases with scalars between −1 and +1, we took the standard score of the mean value, (about zero) and then defined cases that were between ±2 SDs from the mean and the extremes as being “directional.” Cases with values falling within the ±2 SD limits were classified as non-directional. The distribution of PMA directionality of NS neurons by location is shown in Table 1.

**Table 1:** **The distribution of recorded neurons by location**.

**Category**	**Putamen**		**Caudate**		**Bridge**		**Total**	
**Recorded cells**	104	73%	20	14%	18	13%	142	100%
**RFs tested**	81	78%	16	80%	16	89%	113	80%
Cutaneous	7	9%	1	6%	2	13%	10	7%
Deep	27	33%	3	19%	4	25%	34	24%
No Clear RF	47	58%	12	75%	10	62%	69	49%
**PMA**								
Cue	11	11%	2	10%	1	6%	14	10%
Movement	85	82%	17	85%	17	94%	119	84%
Intermediate	8	8%	1	5%	0	0%	9	6%
**PMA DIR R Trials**								
Reciprocal	36	35%	6	30%	5	28%	47	13%
Directional	4	4%	0	0%	0	0%	4	3%
Non-Directional	64	62%	14	70%	13	72%	91	64%
**PMA DIR A Trials**								
Reciprocal	35	34%	5	25%	3	17%	43	30%
Directional	2	2%	1	5%	0	0%	3	2%
Non-Directional	67	67%	14	70%	15	83%	96	68%

RF type and RF location. PMA relationship to cue and movement. PMA changes during certain/uncertain “A” and “R” trials.

### Statistical analysis

Analyses of the characteristics of activity-to-movement relationships for different NS neurons were examined for statistical significance using repeated measures ANOVA, including Scheffe's *post hoc* test and *t*-test. The observations were also checked using the nonparametric Mann Whitney *U*-test and Kruskal–Wallis tests to determine the significance levels of the differences in CoDs and ToCs, for groups separated by trial type (regular or after), go-cue (visual or vibratory), and movement direction (flexion or extension).

## Results

### Database

A total of 236 recorded neurons, 142 (~60%) were selected, because each neuron: (i) had activity changes (PMA) following the vibratory or visual go-cue onset and prior to MOS, (ii) had a PMA firing rate that was at least 3 SDs different from the Bkg activity firing rate, and (iii) was held long enough to record at least 25 trials for each movement direction. Of these, 102/142 (~72%) also had a complete set of recordings during visually cued trials. Of the selected NS neurons, 104/142 (~73%) neurons were located in putamen, 20/142 (~14%) in the caudate nucleus, and 18/142 (~13%) in the cellular bridges in between these structures. We eliminated 3/142 neurons from consideration because their activity was completely suppressed during the PMA interval. Also, the activity of 3/142 neurons during flexion movements and of 7/142 neurons during extension movements had CoDs outside the range that included ±4 SDs from the distribution of the main population and were thus excluded as outliers. This left for statistical consideration the records of 136/142 (96%) cells for flexion movements and 130/142 (92%) cells for extensions. Receptive fields (RF) were found for 44/142 (~31%) NS neurons; 69/136 (~49%) had no clear RFs and 29/142 (~20%) were not tested. Of the cells exhibiting RFs, 10/44 had cutaneous RFs and 34/44 had deep RFs. Table 1 shows the distribution of NS neurons by location and RF type.

The activity patterns of the striatal neurons could not be defined as a homogeneous group. Separation was required because these neurons had significant differences in their activity-to-movement relationship as a function of trial type, cue, movement direction, and location revealed by factorial ANOVA and Scheffe's *post hoc* comparisons. Therefore, our data were grouped by these variables.

### PMA and movement initiation

Significant changes in neuronal activity, defined as a deviation of at least ±3 SDs from the baseline that lasted more than 40 ms (see Nelson and Douglas, 1989; Gardiner and Nelson, 1992) were commonly related either to the go-cue or to the movement. Neuronal firing changes were considered to be cue-related if they occurred in a consistent temporal relationship with the cue and followed its presentation after a relatively short latency (<100 ms). Of the total number, 14/142 (~10%) had cue-related activity, while 9/142 (~6%) had very early PMA which we termed “intermediate” responses. Each of these also had movement related activity as did the 119/142 (~84%) which show activity changes only in relationship to movement onset (see Table 1 for their distribution by location). Significant changes in firing rate that were time locked with MOS, but that preceded MOS, were considered to be PMA (Gardiner and Nelson, 1992; Lebedev et al., 1994; Lebedev and Nelson, 1995; Opris et al., 2011a). Changes in neuronal firing rates that occurred during the execution of wrist movement (i.e., from MOS until potential reward delivery) were classified as “movement-associated activity.” Movement related cells exhibiting PMA may be involved in either movement initiation or execution, depending on the time of occurrence of their activity changes.

### Neural activity to movement kinematics relationship

Consistent with the motor role of dorsal striatum, the firing rate of a subpopulation of neurons in putamen reflect the encoding of hand kinematics (position, velocity, and acceleration).

#### Examples of differential firing during encoding of hand position

**Figure 4:**
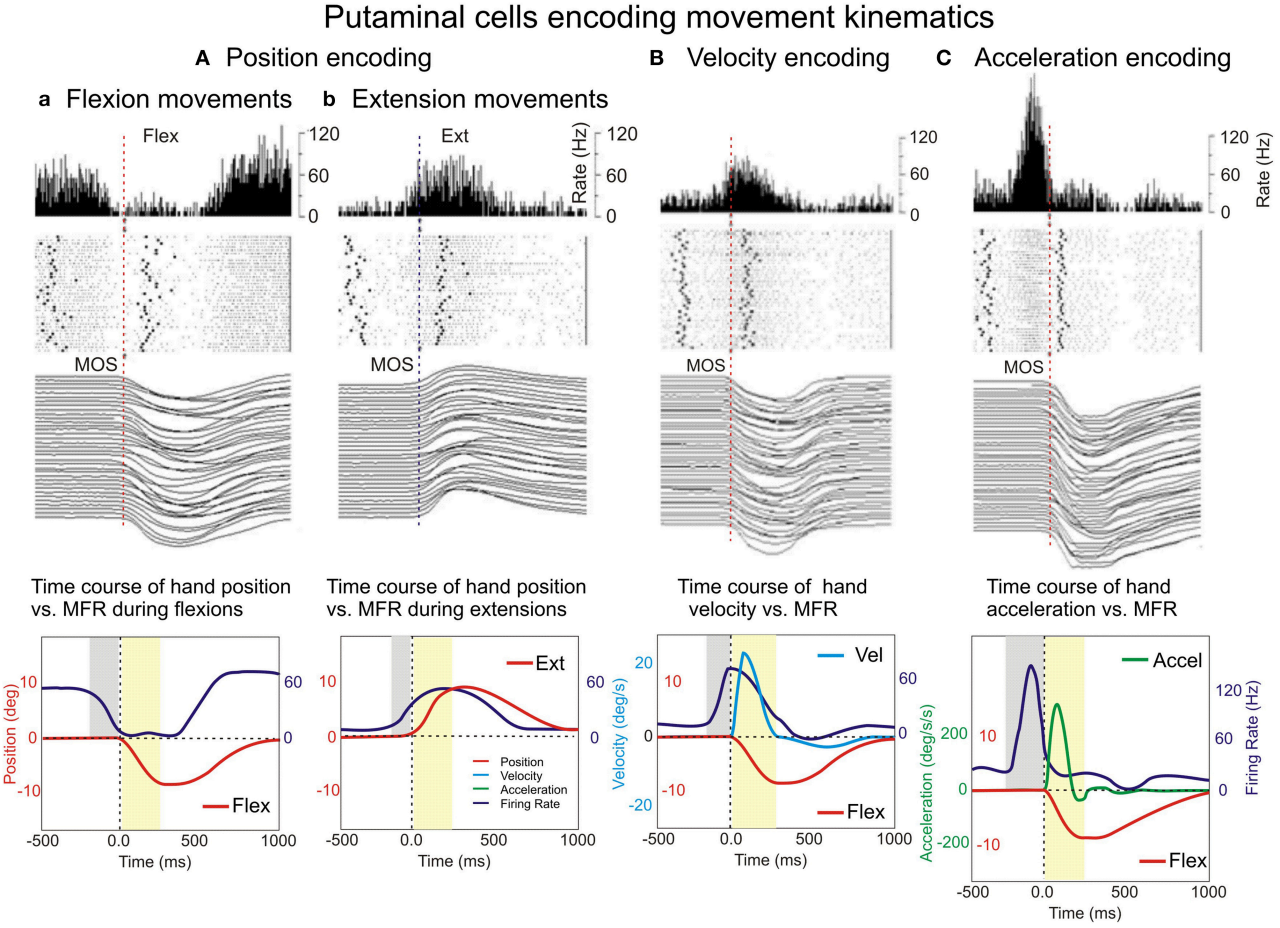
**Examples of encoding the variables of movement kinematics**. Putamen neurons show encoding of hand position (**Aa**, flexions and **b**, for extensions) movement velocity **(B)** and acceleration **(C)**. Peri-event histograms (PEHs), rasters, and movement trajectories were plotted for R flexions and extensions **(Aa,b)**, velocity **(B)**, and acceleration **(C)**. The PEHs of the neuronal activity expressed as mean firing rate (in spikes/s), with a bin size equal to 5 ms and a raster display. In the raster display, rows represent individual trials, dots represent single spikes, while the left and right bold dots represent vibratory cue onset and reward delivery, respectively.

Figure 4A shows the PMA of a putamen neuron classified as “reciprocal” that exhibits a strong suppression during flexion trials while during extension trials this cell's firing is substantially increased. This differential mean firing rate during flexion and extension movements likely reflects the encoding of hand position in the two opposite movement directions (flexions and extensions). At the population level, the encoding of position occurred in 47/142 (33%) VIB cued neurons during uncertain reward “R” trials and 43/142 (30%) during certain reward “A” trials, and in 32/102 VIS cued neurons during uncertain reward “R” trials and of 38/102 NS neurons during certain reward “A” trials. Only 4 NS neurons were “directional” during “R” trials and three cells were “directional” during “A” trials. A minority of the population changed the type of direction-related activity between “R” and “A” trials.

The non-directional NS cell subpopulation which included the vast majority of the studied neurons (*vibration cues*: 91/142 neurons during “R” trials and 96/142 neurons during “A” trials; *visual cues*: 66/102 neurons during “R” trials and 60/102 neurons during “A” trials) had characteristics consistent with the encoding of the magnitude of the position (distance from the center) shown for the population, as a whole, in Tables 1, 2. This type of non-directional modulation was noticed in most of the cells, regardless of go-cue modality. That is, the sign of the activity changes of these neurons was the same regardless of the direction of the movements that followed the onset of this activity (Gardiner and Nelson, 1992).

**Table 2:** **The means and standard deviations for Bkg and Resp neural activity, premovement onset R2s, time of best regression/correlation ToC, and the coefficients of determination CoD for flexions and extensions trials, under certain vs. uncertain rewards**.

**Go Cue**	**Vibratory**				**Visual**			
**Direction**	**Flexions**		**Extensions**		**Flexions**		**Extensions**	
**Trial Type**	**R Trials**	**A Trials**	**R Trials**	**A Trials**	**R Trials**	**A Trials**	**R Trials**	**A Trials**
Bkg (Hz)	21 ± 14	21 ± 14	20 ± 14	21 ± 13	19 ± 13	20 ± 14	20 ± 14	20 ± 13
Resp (Hz)	25 ± 16	26 ± 16	24 ± 14	25 ± 14	24 ± 15	24 ± 16	24 ± 14	24 ± 14
R2 (ms)	187 ± 74	160 ± 54	173 ± 52	158 ± 49	228 ± 70	215 ± 60	185 ± 42	185 ± 50
ToC (ms)	88 ± 70	84 ± 60	73 ± 62	68 ± 58	154 ± 59	161 ± 52	121 ± 51	121 ± 46
CoD	0.871 ± 0.054	0.888 ± 0.056	0.861 ± 0.054	0.867 ± 0.054	0.902 ± 0.04	0.908 ± 0.033	0.903 ± 0.039	0.908 ± 0.033

Comparison between values for certain vs. uncertain rewards, in respectively, “A” and “R” trials. Statistical significance was determined using repeated measures ANOVA, including Scheffe's post hoc test and t-test (See Text for abbreviations).

#### Examples of differential firing with hand velocity and acceleration encoding

In Figure 4B is shown the PMA of a putamen neuron, cued by vibratory stimuli (“A” trials) with firing rate mimicking the velocity profile (on the left). On the other hand, Figure 4C shows a sharper PMA modulation of a putamen neuron, cued by visual stimuli (“A” trials) with firing rate mimicking the acceleration profile (on the right). It is noticeable a sharper firing peak encoding acceleration that is accompanied by a deeper inhibition trough than in the case of cell encoding velocity. Bkg and Resp firing rates (Figures 4B,C) were again slightly higher during “A” trials (not shown) than in “R” trials.

### Neural activity-to-movement kinematics analysis

**Figure 5:**
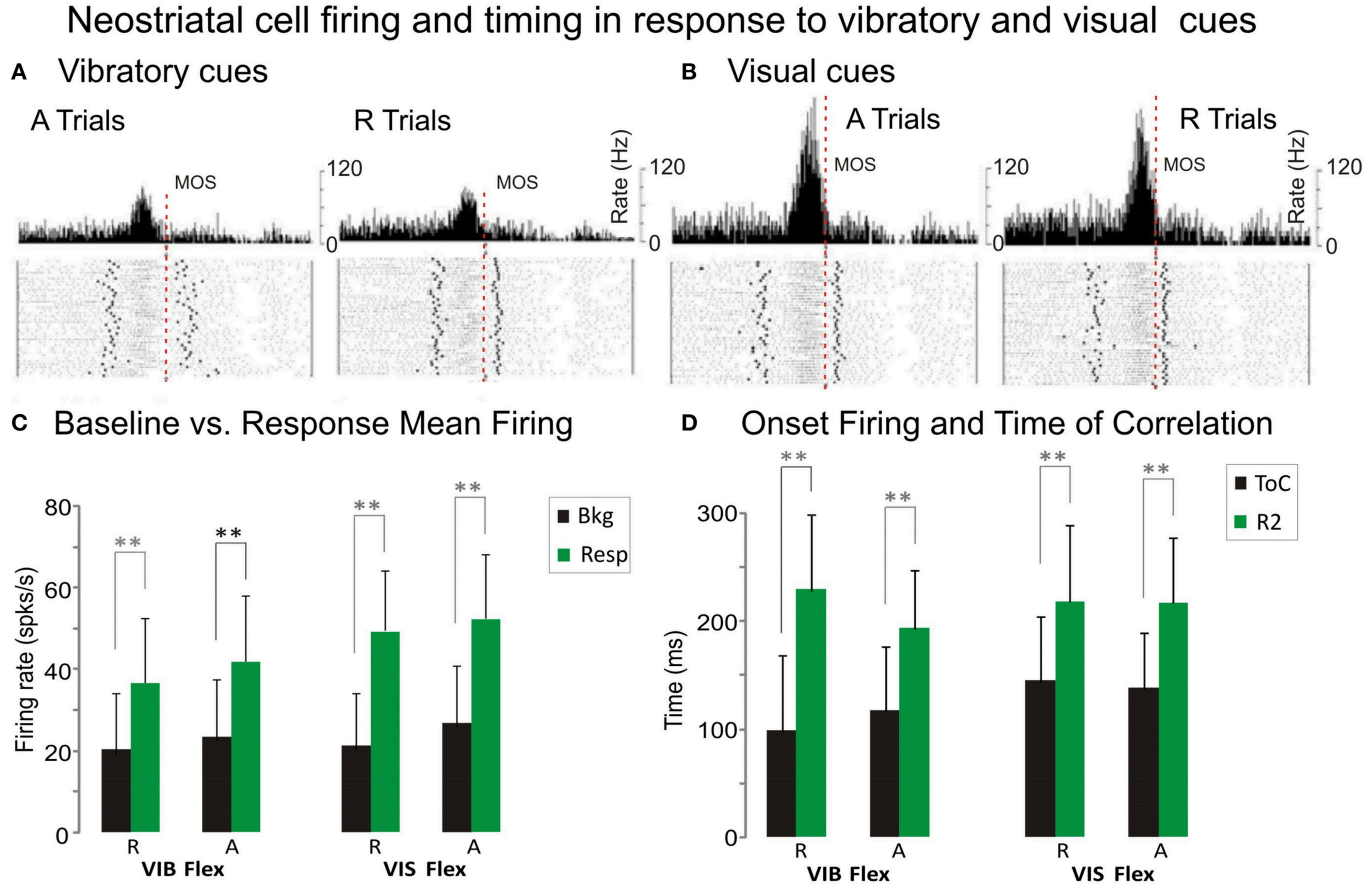
**Premovement firing and timing. (A,B)** Example of putaminal cell that exhibited PMA under vibratory **(A)** and visual **(B)** stimuli. Each histogram illustrates neuronal activity expressed as mean firing rate together with raster displays aligned on MOS. The left panels display the NS activity during “A” flexion trials and the right panels represent the activity during “R” flexion trials. Wrist position traces for each trial are presented at the bottom of each panel. Coefficients of Determination (CoDs) which quantify the best relationship, are: 0.920 ± 0.044 during R trials and 0.941 ± 0.024 during “A” trials (Figure 4A). CoDs for R and A trials are: 0.942 ± 0.021 and 0.954 ± 0.021, (Figure 4B) respectively. This cell exhibited increased discharge during flexion movements that occurred well before MOS. Baseline firing BKG and RESP are higher during trials following withheld rewards than during R trials. PMA onsets together with ToCs suggest that the cell may have been involved in movement initiation. **(C)** Comparison of premovement mean firing rates of NS cells during baseline BKG and response RESP for flexions and extensions under vibratory and visual cues. **(D)** Comparison of the premovement activity timing for response onsets and of best correlation time ToC for flexions and extensions under vibratory and visual cues. Statistical significance was determined using repeated measures. **p < 0.001 ANOVA, including Scheffe's *post hoc* test and *t*-test.

The regression analysis described above was used to investigate changes in NS activity associated with movement initiation under two conditions. Comparisons were made between trials with uncertain rewards (“R” trials) and trials with certain rewards (“A” trials). We illustrate the activity patterns of a representative putamen neuron recorded during vibratory go-cue trials, in Figure 5A, and the pattern of the same neuron recorded during visual go-cue trials, in Figure 5B. The records of “R” trials (left panel, Figure 5A) and “A” trials (right panel, Figure 5A) are illustrated for flexion movements. This cell exhibited an increased discharge, before MOS (Figure 5A), and thus PMA preceding wrist flexion (position traces displayed for both “R” and “A” conditions). The neuron was more active in the blocks of vibratory cued “A” trials than during R trials (left panel, Figure 5A). Bkg and Resp were also higher when rewards were certain. In addition to PMA changes associated with unpredictable reward conditions, this putamen neuron's activity became better correlated with animal's movement. This is accompanied by changes in the average CoDs, which were greater during “A” trials than during “R” trials (0.941 vs. 0.920). The time occurrence of the best correlation (ToC) was ~117 ms before MOS during “A” trials and ~99 ms, during regular reward.

This same putamen neuron, whose visually cued trial records are shown in Figure 5B exhibited a higher firing rate during the “A” trials for flexion movement under this cueing condition, as well. The Bkg and Resp are higher during “A” trials (26.8 and 52.2 spks/s) than during regularly “R” rewarded ones (21.3 and 49.3 spks/s). The PMA changes occur quite early, before MOS. The increase in CoDs in “A” trials, as compared with “R” trials, indicates that PMA becomes better correlated with the movement after withheld rewards (0.954 vs. 0.942). Comparing the neuronal activity during visual go-cue trials with vibratory go-cue trials, one can note an average increase (of 10 spks/s) in the mean firing rate of PMA that occurred during the former. However, average CoDs during visually cued A trials were greater than during cued trials (0.954 vs. 0.941).

At the population level, NS firing was significantly higher in the Resp vs. Bkg conditions for the majority of cells (*p* < 0001, *post hoc*). However, when comparing mean firing rate across trial type, go-cue type or movement direction the effects varied (see Table 2). Bkg and Resp firing rates were slightly higher during “A” vs. “R” trials (Figure 5C) and during VIS vs. VIB trials (*P* < 0.001, *post hoc*; *n* = 91 vs. 66).

Additional information about the timing of this relationship can be derived from the examination of the time of occurrence of best CoDs i.e., (ToCs), which indicate the time of best CoDs at either MOS or AOS, respectively (see Table 2). A comparison of PMA onsets and ToCs for vibratory cued trials is shown in Figure 5D. The ToCs during visually cued trials occur much earlier than during vibratory cue trials. The ToCs in “A” vs. “R” trials for flexion movements were significantly different only with visual go-cues (see Table 2). There was also a statistical significance in the ToCs for flexions and extensions triggered by visual cues (not shown). Although we were unable to distinguish between direction and load effects (since monkeys always performed the movements against a load which assisted wrist extensions and opposed flexions), the effects of direction and load were evident through the fact that interfered with each other.

PMA onsets for single trials were measured relative to two events: go-cue onsets (COS) and movement onsets (MOS; see Figure 1). R1s, measured from COS, during vibrotactile stimulation, for both flexion and extension movements were significantly shorter in the A trials (see Table 2). R2s, measured from MOS, were calculated by subtracting the R1 from the RT (Figure 1). Flexion movements had R2s that were significantly shorter during “A” trials regardless of go-cue modality. Thus, PMA during vibratory cued trials started ~27 ms closer to MOS in the “A” trials when compared with “R” trials. During visually cued flexion trials, it started closer to MOS by ~13 ms. R2s were significantly different during visually cued flexion and extension movements, but not different during vibratory cued trials.

Statistical analyses of the activity-movement kinematics relation under unpredictable reward conditions are presented in Table 2. We have considered the trial type as a repeated measure and split each of the PMA-related variables by the go cue type and movement direction. Significant differences in the variables between conditions (“R” and “A” trials) were obtained by repeated measures ANOVA.

### Coefficients of determination

**Figure 6:**
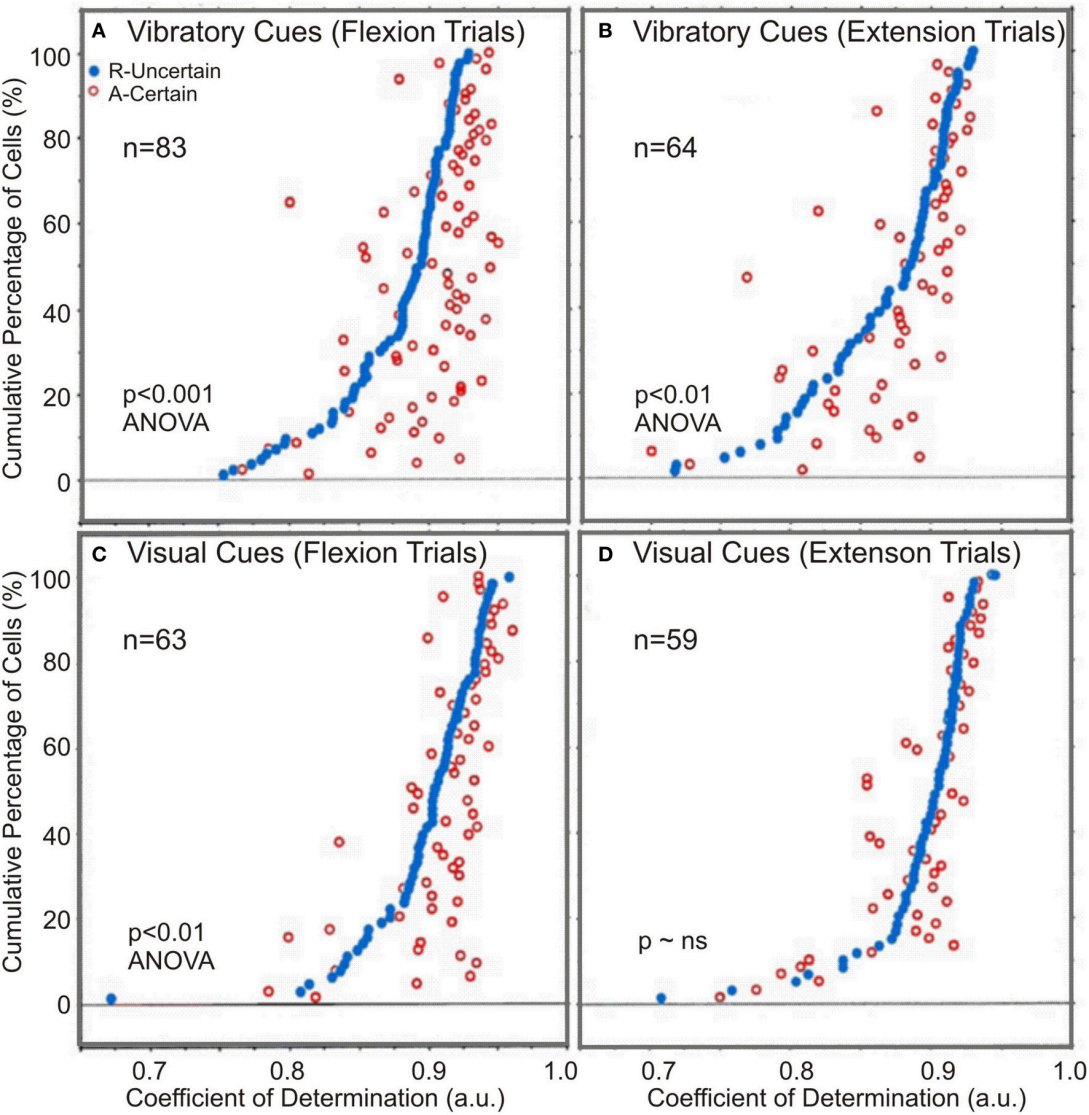
**The comparison of regression coefficients**. Paired coefficients of determination, CoDs, for both vibratory and visually cued trials: **(A,B)** Certain “A” and uncertain “R” rewarded trials for vibratory cued trials and **(C,D)** for visually cued trials. In both instances, CoDs of the uncertain R trials (black dots) were arranged in ascending order from left to right. The certain trial CoDs corresponding to each uncertain trial CoDs were plotted at the appropriate height. Open dots to the right of the black dots indicate instances where the neuronal activity was better related (CoDs were greater) for movements made in the certain reward A trials.

A measure of the correlation's strength in “R” and “A” trials is provided by the average CoDs. In general, the average CoDs showed a tight relationship between NS neuronal activity and monkey's kinematics (see Table 2). Flexion movement CoDs were significantly greater during “A” trials than during “R” trials (*p* < 0.01; ANOVA) regardless of the modality of go cues (see Table 2). The occurrence of enhanced activity-to-movement correlation in trials following withheld rewards is illustrated in Figure 6. Cumulative sum plots of CoDs for “R” and “A” trials show a consistent shift to the right for the “A” trials (*p* < 0.001 for vibratory flexions; *p* < 0.01 for vibratory extensions; *p* < 0.01 for visual flexions; *p* ~ ns for visual extensions; ANOVA), independent of go-cue modality.

There was also a directional effect, in that CoDs after withheld rewards were significantly greater during flexion trials than during extension trials (*p* < 0.001 for flexions; *p* < 0.01, for extensions, ANOVA), for both vibratory cued trials and visually cued trials (Table 2). In general, there was a better correlation of NS neuronal activity with kinematics in visual go-cue trials compared with that in vibratory go-cue trials (*p* < 0.001 for flexions). The average CoDs for visually cued flexions during “A” trials were greater during vibratory go-cued flexion “A” trials.

Given the existence of the activity-to-movement relationship (measured by the CoD) during trials following withheld rewards, we noticed three distinct effects: first, an effect of unpredictable reward which results in a better activity-to-movement correlation for certain rewarded “A” trials than for “R” trials; second, a directional effect which results in a better correlation for flexion than for extension movements for both cues, and third, a modality effect as indicated by better relationship for visually cued as compared with vibratory cued movements.

### Electromyographic activity

**Figure 7:**
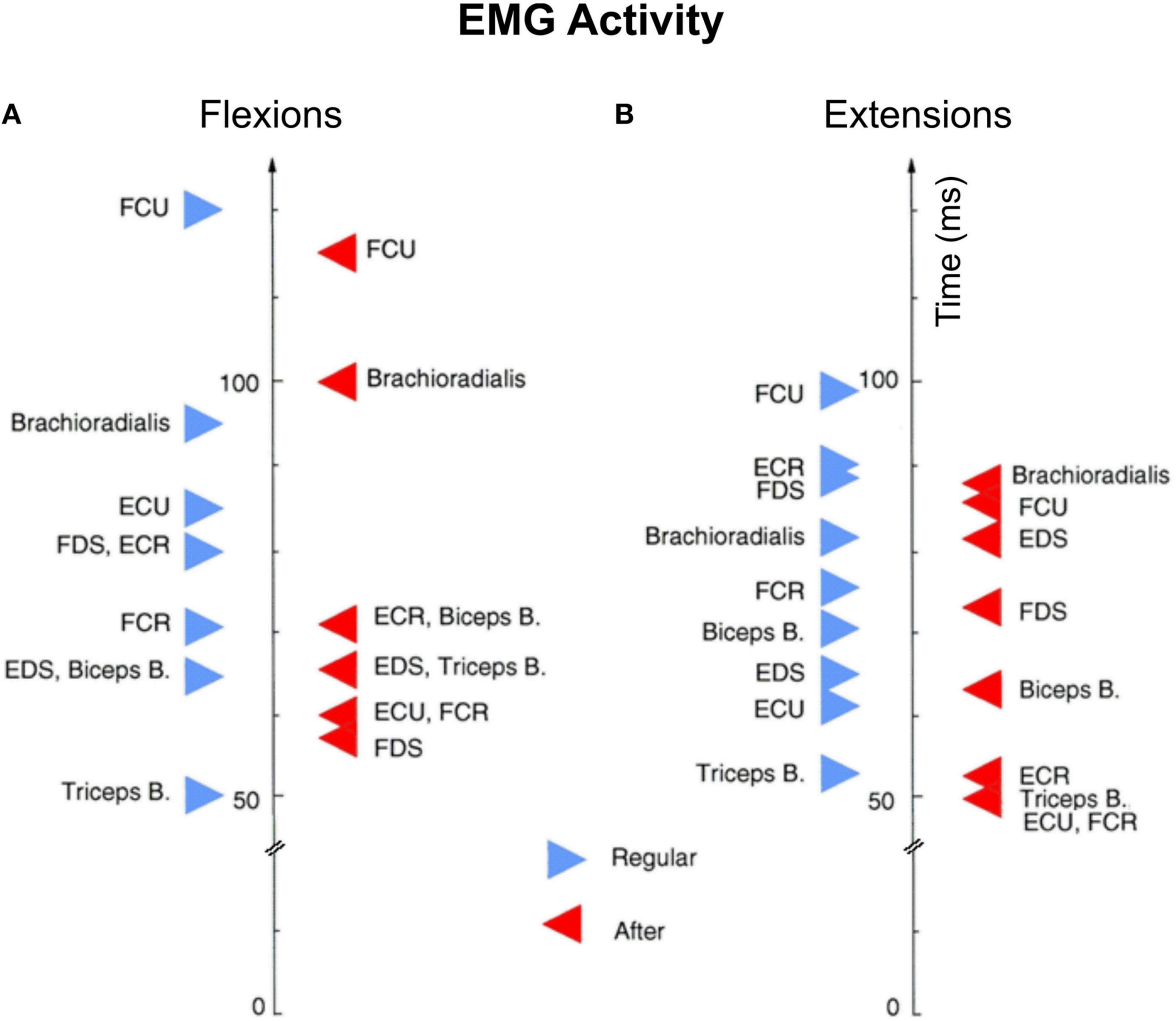
**Average onsets of electromyographic (EMG) activity**. EMG activity between flexion **(A)** and extension **(B)** movements for both certain and uncertain reward trials. Each triangle (red for certain and blue for uncertain reward trials) represents the average EMG onset for at least 40 trials recorded consecutively. Biceps B, biceps brachii; BR, brachioradialis; ECR, extensor carpi radialis; ECU, extensor carpi ulnaris; EDS, extensor digitorum superficialis; FCR, flexorcarpi radialis; FDS, flexor digitorum superficialis; FCU, flexor carpi ulnaris; FDS, flexor digitorium superficialis; Triceps B, triceps brachii.

We compared average onsets of electromyographic (EMG) activity between flexion (Figure 7A) and extension (Figure 7B) movements for both “Regular” and “After” trials. With few exceptions, for most muscles the EMG activity starts earlier in the “After” trials than in “Regular” trials. Brachioradialis has the opposite trend in the “After” vs. “Regular” trials for both flexions and extensions. All together the EMG and behavioral measures seem to have a consistent agreement with NS pre-movement neural activity supporting the hypothesis that under certain rewards NS neuronal activity becomes better correlated to movement kinematics.

## Discussion

In the present study we recorded the activity of neostriatal neurons in two rhesus monkeys performing wrist movements in a pseudo-random reward task. We examined the relationship between PMA and kinematic variables (position, velocity, and acceleration) under three conditions: (a) reward contingency, (b) vibratory vs. visual go-cues, and (c) flexions vs. extensions movements (Opris et al., 2011a).

The results of this study indicate that NS neurons have PMA which is functionally correlated with movement kinematics. This correlation varies as a function of reward contingency (unpredictable vs. predictable delivery of reward for correct performance). Our goal was to determine whether there is a relationship between NS PMA and movement kinematics and to quantify this relationship in terms of coefficients of determination (CoDs) and relationship occurrence times (ToCs). These parameters varied significantly not only as a function of rewarding conditions, but also with variations in the modality of sensory triggering stimuli and with the direction of movement. In addition, we found that reaction times RTs and PMA onsets are also significantly different as a function of reward schedule and go-cue modality. These results suggest that it is likely that the NS is involved in sensorimotor-related activity that is combined with attentional, decision, and motivational influences (Schultz, 1997, 2010; Schultz et al., 1997; Opris and Bruce, 2005; Samejima et al., 2005).

### Neural firing to movement correlation under unpredictable task

The statistically significant differences in CoDs for “R” and “A” trials may be related to attention and motivational factors associated with reward (Watanabe, 1996; Ueda and Kimura, 2003). Changes in the predictability of reward delivery, assumed to occur after withheld rewards, are accompanied by increases in CoDs and decreases in reaction times. Furthermore, changes in the predictability of reward delivery are accompanied by increases in CoDs, suggesting that the changes in CoDs are related to an animal's increased attention.

Differences in CoDs as a function of go-cue modality may be related to the gating of somatosensory inputs that often accompanies sensory-triggered movements. Our results indicate that the CoDs associated with NS neural activity during vibratory cued trials have significantly lower values as compared with those occurring during visual go-cued trials. Vibratory stimuli are one type of peripheral input to SI neurons that may be gated before active movements (Lebedev and Nelson, 1995). Because somatosensory cortex projects extensively to the neostriatum (see Parent and Hazrati, 1995 for review), it seems reasonable to suggest that gating in the cortex could ultimately result in modulation of NS activity. Changes in CoDs as a function of go-cue modality may be a reflection of alterations in cortical inputs to NS, as well as modulatory effects by dopaminergic systems (Lebedev and Nelson, 1995; Kiyatkin and Rebec, 1996). It has been suggested that changes in behavioral motivation may be mediated by dopaminergic neurons (Apicella et al., 1991; Schultz et al., 1992, 1993). Therefore, the dopaminergic pathways arising from the pars compacta of substantia nigra (SNc) and projecting to dorsal striatum (Lynd-Balta and Haber, 1994; Parent and Hazrati, 1995) may be involved in the motivational process underlying the learning and maintenance of goal driven behavior (Mirenowicz and Schultz, 1994; Schultz et al., 1997).

Statistically significant differences in CoDs between trials involving flexion and extension movements may be due to the fact that the movements were performed against a load which assisted wrist extensions and opposed flexions. Flexion trials had CoDs that were significantly higher than those during extension movements. The presence of the load may add a small contribution to the relationship during flexion movements or subtract that contribution when moving with the load (Liles, 1985; Alexander and Crutcher, 1990; Gardiner and Nelson, 1992). Sensorimotor territories of NS may also show differential firing as a result of cortical load effects, since it has been suggested that there are several parallel pathways of somatotopic input to NS (Alexander et al., 1986; Graybiel et al., 1994). Therefore, the difference in CoDs as a function of movement direction may be influenced by load, the movement direction itself, or both.

### Pre-movement activity and regression times as a function of reward schedule

In general, PMA activity was well correlated with wrist movement variables. The average CoD of the whole NS neuronal population was >0.85 for each modality, movement direction, recording location, and rewarding schedule. During vibratory cued trials which follow withheld rewards, there is a shift in PMA onset toward MOS despite the fact that reaction times become shorter. Schultz and co-workers have demonstrated that leftward shifts in the onset of activity occur in the firing patterns of dopaminergic neurons when tasks become more predictable (Schultz et al., 1992, 1993). The corollary of this would be that if the process was reversed and task requirements become more unpredictable, rightward shifts in activity onsets might be observed as was indeed observed in this study. Similar shifts in PMA were seen by Kimura (1990) when the amount of prior task information about behavioral requirements was varied. In our task, shifts in reaction times and PMA onsets, as shown in Figure 6A, occur after withheld rewards (Kimura et al., 2003; Churchland et al., 2006; Hori et al., 2009). Despite these shifts, the onsets of PMA and ToCs are early enough to suggest that the activity of NS neurons may be associated with the initiation of wrist movements, as well as its execution (Graybiel, 1990; Romo et al., 1992; Nelson et al., 1996). The onset of PMA in NS occurred earlier than did PMA onset for somatosensory cortical neurons when monkeys performed the same task (unpublished observations).

### Functional consequences of unpredictable reward

The mechanisms contributing to sensorimotor integration in the NS, as perhaps indicated by PMA, are not completely understood. However, part of a mechanism necessary for the shaping of NS activity during the initiation and execution of motor behavior has been suggested to involve the dopaminergic (DA) modulation (Surmeier and Kitai, 1997). There are several relevant features of DA modulation. DA modulation is a dynamic process that, depending on the level of membrane depolarization, causes an increase in the NS neuron firing rate if the membrane is in an “up” state or a decrease in firing rate if the membrane is in the “down” state (Hernández-López et al., 1997).

According to Berns et al. (1997) the withholding of rewards may be considered to be a context violation which may be thought of as a breach in expectation. These authors suggest that ventral striatum becomes activated when contexts are violated by stimuli that appear unexpectedly. It is reasonable to assume that the absence of reward, when it is expected, is itself, an unexpected stimulus. Dopamine neurons become activated during unpredictable behavioral conditions, thereby providing contrast to previously fully predicted stimuli (Mirenowicz and Schultz, 1994). As a working hypothesis, we suggest that the dopamine system, through its projections to NS, may influence neuronal activity (Schultz and Romo, 1988; Pasquereau et al., 2007), resulting in an increase of covariant relationship between PMA neuronal activity and movement variables (Nelson et al., 1996). Moreover, learning theories suggest that the learning process is driven by the unpredictability of reward, and that little or no further learning takes place when reward is entirely predicted (see Schultz et al., 1997 for review). Dealing with unpredictable aspects of behavior seems to involve a higher order processing of information in basal ganglia and require multiple processing channels (Hoover and Strick, 1993).

The results presented in this work are consistent with the view that NS may provide an “interface” between sensorimotor, limbic and association subcortical territories and cortical areas involved in higher brain functions such as motivation, attention, and memory (Evarts and Wise, 1984; Schultz et al., 1997; Opris et al., 2011a,b, 2013; Santos et al., 2014). The complete understanding of these functions requires more experimental and theoretical work. However, it is likely that cerebral cortex and basal ganglia structures utilize different modular subsets at any given time during highly dynamic processing involved in sensorimotor integration (Evarts et al., 1984).

## Summary

When monkeys are rewarded pseudo-randomly (75% of the correct trials) in a task requiring either vibratory or visually-cued wrist movements, neostriatal PMA is tightly correlated with movement kinematics. There are two different modes of this activity-to-movement correlation. The correlation between neuronal activity and movement, during flexion trials but not extension trials, was higher in trials with certain rewards as compared to the trials with uncertain rewards. The improvement in the activity-to-movement correlation was accompanied by shifts in PMA onsets so that they occurred closer to movement onset and by shorter reaction times (Opris et al., 2011a). Thus, the changes in the predictability of behavioral requirements are reflected in the correlation between neostriatal PMA and the kinematics of wrist movements. These observations are consistent with the hypothesized modulation of neostriatal activity by the dopaminergic systems during certain vs. uncertain behavioral situations.

## Author contributions

IO performed the data analyses and wrote the manuscript. ML collected the data. RN supervised all aspects of the research.

### Conflict of interest statement

The authors declare that the research was conducted in the absence of any commercial or financial relationships that could be construed as a potential conflict of interest.
